# Orchestrating Extracellular Vesicle With Dual Reporters for Imaging and Capturing in Mammalian Cell Culture

**DOI:** 10.3389/fmolb.2021.680580

**Published:** 2021-06-18

**Authors:** Daniel Levy, Mai Anh Do, Jiayi Zhang, Annie Brown, Biao Lu

**Affiliations:** Department of Bioengineering, School of Engineering, Santa Clara University, Santa Clara, CA, United States

**Keywords:** nanotechnology, VSVG, CD63, exosome, extracellular vesicle (EV)

## Abstract

**Background:** Recent technological advancements have enabled live-cell imaging of intracellular organelles to monitor their biogenesis in mammalian cells. However, applying this method to gain insight into extracellular organelles, such as extracellular vesicles (EVs), presents unique challenges that require special considerations in design and engineering.

**Results:** We have developed a dual-reporter system that combines genetic fusion, fluorescence microcopy and magnetic beads capture of EVs to study the biogenesis of EVs in mammalian cell cultures. First, we genetically produced a series of reporters by fusing a green fluorescent protein (GFP) and an affinity peptide (6xHis), with either the endogenous transmembrane protein, CD63, or EVs targeting vesicular stomatitis viral glycoprotein (VSVG). Transfection of these reporters into human 293T cells resulted in expression and integration of these reporters into pre-exosome compartments, which were subsequently released into the culture medium. Confocal imaging and nano-particle tracking analysis demonstrated that EVs were appropriately labeled and exhibited a single dominant peak in the 80–110 nm size range, indicating that isolated EVs were comprised of micro-vesicles and/or exosome subpopulations. Incubation of isolated EVs with nickel-coated magnetic beads resulted in successful capture of GFP-positive EVs. Finally, addition of EVs into culture medium was able to reveal the cellular uptake of GFP-labeled EVs by recipient cells. Taken together, our dual-reporter system provides a powerful method for both monitoring and capturing of EVs in mammalian cell culture systems.

**Conclusion:** A dual-reporter system provides a robust tool to study the life cycle of EVs in mammalian cells from biogenesis and excretion to cellular uptake.

## Introduction

Genetic fusion of organelle-targeting scaffolds with fluorescent proteins provides an important tool for studying the biogenesis and regulation of intracellular structures such as mitochondria (Gilkerson et al., 2000), the ER-Golgi apparatus (Lippincott-Schwartz et al., 2001), and autophagosomes (Kimura et al., 2007). When introduced into living mammalian cells, these molecular reporters can display individual organelles and allow live-cell trafficking of organelles in real-time, thereby gaining valuable insights into the mechanism of their biogenesis and regulation in potential physiological and pathological conditions (Tsien, 1998). However, existing design platforms are skewed toward the study of intracellular organelles, which may be insufficient for the display of extracellular organelles such as EVs. For example, after releasing into culture medium, the secreted EVs are quickly diluted and become undetectable with imaging microscopes. Therefore, an additional affinity tag that enables capturing of secreted EVs is critical for continued studying the full life cycles of EVs, including molecular characterizations and subsequent uptakes by recipient cells.

EVs are lipid-bilayer-enclosed nanovesicles that are secreted by almost all types of mammalian cells (EL Andaloussi et al., 2013; Colombo et al., 2014). A number of reports suggest that EVs carry a wide variety of bioactive cargos, including proteins, nucleic acids, and lipids (Valadi et al., 2007; Simpson et al., 2008; Mittelbrunn and Sánchez-Madrid, 2012; Sung et al., 2015). These EV-associated biomolecules play a critical role in regulating diverse natural processes within human body as well as pathological pathways such as viral infection (Meckes and Raab-Traub, 2011; Ramakrishnaiah et al., 2013), immune response (Gutiérrez-Vázquez et al., 2013), neurodegeneration (Kalani et al., 2014; Pulliam et al., 2019), cancer progression and metastasis (Peinado et al., 2012; Melo et al., 2015). As the study of EVs remains an evolving field, EVs hold great promise for disease diagnosis as well as drug delivery and therapy (Barile and Vassalli, 2017; Kamerkar et al., 2017; Riazifar et al., 2017; Do et al., 2019; Duong et al., 2019). However, a lack of robust research tools has hampered the ability to study EV biogenesis and to find therapeutic solutions (Shahabipour et al., 2016; Sterzenbach et al., 2017). Despite a few molecular methods for EVs imaging and tracking, improvements to enhance the specificity, stability and effectiveness of EVs labeling are still imminent (Petros and DeSimone, 2010; Marcus and Leonard, 2013; Hung and Leonard, 2015; Ingato et al., 2016).

Here, we report the development of a dual-reporter system, which enables both live-cell imaging and specific capture of EVs from mammalian cell cultures. By fusing EVs-associated transmembrane proteins, CD63 or VSVG with both a GFP and an affinity peptide (6xHis tag), we are able to topologically display 6xHis on the outer membrane while enclosing GFP within the EVs lumen. The remarkable feature of this systems uses transmembrane protein as the stable anchoring scaffold, while displaying 6xHis tag at the outer surface for affinity purification and protecting GFP inside from potential degradation. By transfection study, we showed that both reporters could integrate into the EVs membrane and the modified EVs could ultimately be released into the cell culture medium. Isolation and characterization of modified EVs further demonstrated that they were properly modified and could be captured *via* Nickel-coated magnetic beads. Finally, these modified EVs enabled live-cell imaging to show the uptake process of EVs by various recipient cells.

## Materials and Methods

### Materials

Dulbecco’s Minimal Essential Medium (DMEM), fetal bovine serum (FBS), GlutaMax, and penicillin/streptomycin were obtained from Life Technologies (Grand Island, NY). FuGENE6 transfection reagent was from Promega Corp (Madison, WI). Human embryonic kidney cells (293T) were from Alstem (Richmond, CA). Human glioblastoma cells (U87), and human liver cancer cells (HepG2) were from ATCC (Manassas, VA). ExoQuick-TC and XPACK-RFP were from System Biosciences (SBI, Palo Alto, CA). His-tag isolation and pulldown Dynabeads were purchased from Invitrogen (Carlsbad, CA).

### Vector Construction

Expression vectors for GFP tagged full-length CD63 and VSVG were constructed as previously reported (Stickney et al., 2016; Meyer et al., 2017). For the addition of a 6xHis tag, DNA sequences encoding the 6xHis-tag were chemically synthesized and subsequently cloned into CD63-GFP by replacing the amino-acid (aa) coding sequences of CD63-GFP (aa 2–62 at its N-terminus) (Stickney et al., 2016) or inserting it immediately after the signal peptide sequences of VSVG-GFP (Meyer et al., 2017). The fidelity of the final constructs was confirmed by double-stranded DNA sequencing. Protein sequences of the dual-tagged chimeric proteins were provided in Supplementary file S1 (Supplementary sequences).

### Cell Culture and Transfection

Human 293T, U87, and HepG2 cells were maintained in high-glucose DMEM supplemented with 10% fetal bovine serum, 2 mM GlutaMax and 100 U/ml penicillin-streptomycin. All transfections were performed in six-well culture plates unless otherwise stated. Typically, cells were grown to ∼40–50% confluency before transfection by addition of plasmid DNA (1–2 µg/well) mixed with either Lipofectamine 2000 or FuGene6 transfection reagents as previously reported. 48 h post-transfection, cells were replaced with fresh culture medium and cultured for an additional 72 h.

### EVs Isolation

Isolation of EVs from the conditioned medium was performed as previously reported (Do et al., 2019). Briefly, following transfection with plasmid DNA for 24 h, 293T cells were switched to serum-free UltraCulture medium. After an additional 48 h in culture, the conditioned medium was collected, centrifuged, and filtered through a 0.22 um filter to remove cell debris and large vesicles. The filtered medium containing primarily exosomes and microvesicles was mixed with the exosome-precipitation solution, ExoQuick-TC and incubated at 4°C overnight. The exosome-ExoQuick solution was pelleted by another round of centrifugation at 3,000 rpm for 90 min. The resulting pellet was resuspended in a phosphate buffer solution for further analysis or stored at −80°C for future use.

### Nanoparticle Tracking Analysis

Nanoparticle Tracking Analysis (NTA) employs light scattering technology and Brownian motion of nanoparticles for sizing determination in an aqueous condition. Typically, 1 ml of a diluted EV preparation was subjected to laser light scattering study. Three recordings of 60 s per sample was obtained using a NanoSight LM10 instrument with a 405 nm and 60 mV laser source. Size distribution of EVs was analyzed and graphed by the NTA software.

### Affinity Capturing of EVs

EVs preparations were mixed with Nickel-coated magnetic Dynabeads at a ratio of 40 μg of EVs protein per 100 μl of beads. The mixture was incubated overnight at 4°C on a rotating shaker. Beads were then washed three times with 500 μl of PBS−0.001% Tween. Bead-bound EVs samples were resuspended in 50 μl of PBS before 1–2 μl was applied to glass slides for confocal imaging at 63× magnification.

### EVs Uptake

EVs uptake assays were performed as previously reported (Do et al., 2019). Briefly, recipient cells were seeded and incubated in FBS-supplemented medium overnight before switching to FBS-free UltraCulture medium. Modified EVs isolated from transfected 293T conditioned medium were added to recipient cultured cells to allow for uptake. At indicated time-points, both fluorescent and phase-contrast images were recorded to demonstrate intracellular distribution of internalized fluorescent EVs.

### Fluorescence and Confocal Microscopy

Images of live cells or fluorescently labeled EVs were recorded using either an Olympus fluorescence microscope or Leica TCS SP8 confocal microscope. To demonstrate intracellular localization of fusion proteins, both fluorescent and transmitted light images from the same field were recorded. Image adjustments such as brightness and contrast were applied to the entire image frame using instrument software.

## Results and Discussions

### Design and Construction of Dual-Reporters for Both Monitoring and Capture of EVs in Mammalian Cell Cultures

**FIGURE 1:**
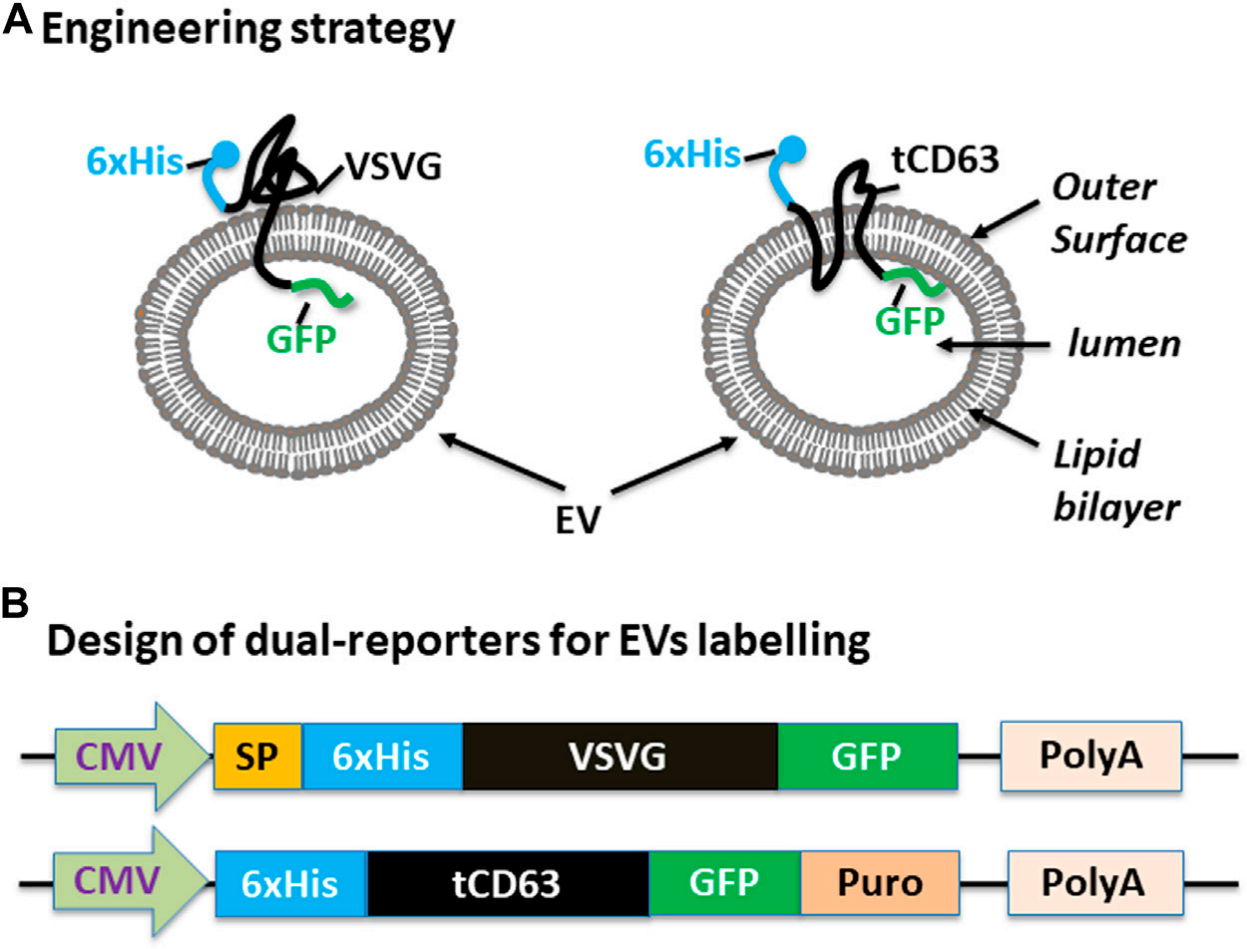
Design and construction of dual-tagged fusion protein for EVs imaging and capturing in human cells. **(A)** Engineering strategy. EVs-membrane anchoring proteins CD63 and VSVG are genetically fused with 6xHis tag and GFP. The affinity 6xHis tag is placed at the N-terminus, on the outer surface of EVs, while the GFP and/or Puromycin reporter is placed at the C-terminus, inside the lumen of EVs. **(B)** Design of the EV-targeted fusion reporters. The coding sequences of the dual-tagged fusion proteins are under the control of a cytomegalovirus (CMV) promoter and terminated by a poly-adenylation signal sequence. When introduced into mammalian cells, these genetic expression constructs will produce fusion proteins that target and integrate into EVs, therefore enabling live cell imaging and capture of EVs from those cells. Color codes: blue (6xHis); black (VSVG/CD63); green (GFP).

We have previously established robust methods to display functional peptides on the surface of EVs using either VSVG or CD63 as the EV-targeting scaffold (Stickney et al., 2016; Meyer et al., 2017). In the present study, we used them to present two reporters (GFP and 6xHis) for better studying the biogenesis of EVs. To achieve this goal, we elected to present the 6xHis (for affinity capturing) at the outer surface while the GFP (for molecular imaging) at the inner surface (Figure 1A). To ensure correct membrane topology, we elected to insert the 6xHis peptide immediately after the signal peptide (SP) of VSVG. For CD63, we chose to delete the N-terminal transmembrane domain of the full-length CD63 and place the 6xHis at the N-terminus, resulting in 6xHis-tCD63. This truncated CD63 retains its ability in EV-targeting, but allows the 6-His situated at the outer surface (Curley et al., 2020). To add GFP reporter, we fused GFP or GFP-Puro at the C-terminus so the imaging GFP will reside in the luminal side of EVs. Finally, we cloned these fusion genes between a CMV mammalian promoter and Poly-A so they can be readily expressed in mammalian cells (Figure 1B). For design details, we provided the amino-acid sequences of these dual-reporters (Supplementary Sequences **)**. Compared to single reporters, our new dual-reporters have several important improvements: 1) addition of 6xHis will facilitate the capturing and isolation of secreted EVs, an important tool study and tracking EVs when they are secreted into extracellular environment (Peterson et al., 2015). When combined with other existing technologies, it may enable high throughput and on-chip analysis; 2) The use of either CD63 or VSVG as the EV-targeting scaffold will provide the flexibility to display two peptide reporters separately at two terminal ends, sterically favoring the bind of 6xHis to Nickel-beads and protecting GFP from potential degradation. 3) Our transmembrane scaffold (VSVG or CD63) are more stable than those of existing lipid anchors, which attach to surface of EVs *via* a non-specific fatty acid insertion (Shi et al., 2004; Shen et al., 2011); 4) The choice of CD63 and VSVG will broaden potential applications. While the endogenous EV mark CD63 is more appropriate for physiological studies and drug delivery (Curley et al., 2020), the viral VSVG will be suited for virus-host interaction and advanced EV-engineering (Meyer et al., 2017).

### Monitoring EVs Biogenesis in Living Human Cells

**FIGURE 2:**
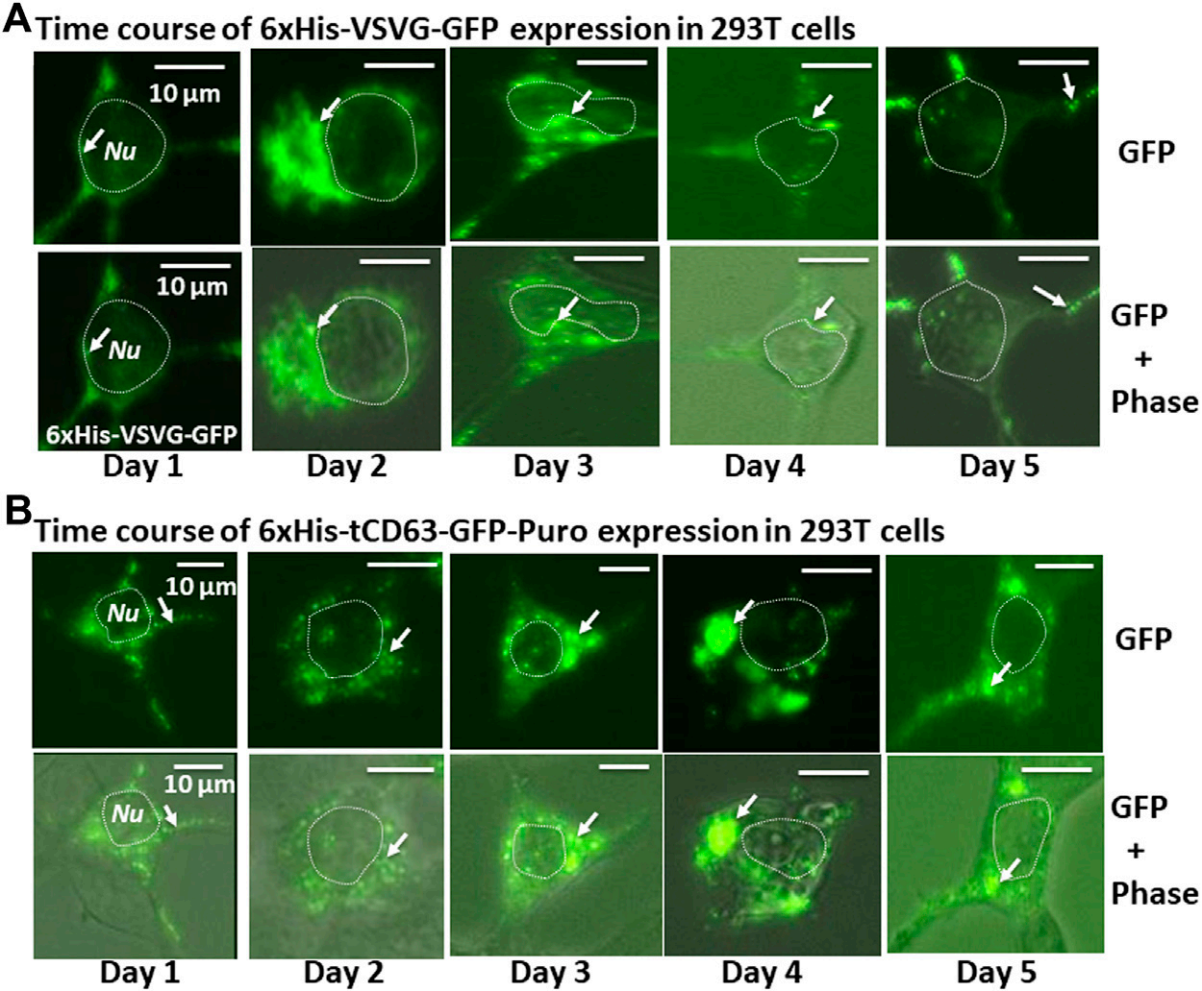
Time-course study of stable labelling and imaging of EVs in living human 293T cells. 293T cells were transiently transfected with either 6xHis-VSVG-GFP. **(A)** or 6xHis-tCD63-GFP-Puro. **(B)**, and imaged after 24 h, and every subsequent day for five consecutive days. The fluorescence signals from these chimeric proteins were recorded to demonstrate expression levels and subcellular distribution. Upper panels are fluorescent images, while the lower panels are overlaid images of green fluorescent images and phase contrast images. Arrows indicate endosome/exosome/MVB structures. Dotted circles indicate cell nuclei (*Nu*). Scale bar, 10 µm.

To examine whether the dual-reporters could be successfully expressed and subsequently integrated into EVs, we transfected 293T cells with the encoded constructs and performed fluorescence imaging for five consecutive days. We found that while GFP appeared as early as 6 h post-transfection (data not shown), they became stronger on Day 1 and persisted up to 5 days for both 6xHis-VSVG-GFP and 6xHis-tCD63-GFP-Puro (Figures 2A,B). These fluorescence signals were mainly located in the cytosol of transfected cells and exhibited discrete green punctates on Day 2 post-transfection (arrows in Figures 2A,B). These results were consistent with endocytic distributions as we previously reported (Meyer et al., 2017; Curley et al., 2020).

To verify above results, we performed similar experiments in other cell lines including human glioblastoma U87 and mouse L929 cells (Supplementary Figure S1 **)**, As expected, both 6xHis-VSVG-GFP (Top panels) and 6xHis-tCD63-GFP-Puro (bottom panels), exhibited a similar patterns of green fluorescence punctates within the cytosol similar to those of transfected 293T cells (arrows in Supplementary Figures S1A, B). These results indicated that our genetic method is robust and can be used in various types of mammalian cells.

**FIGURE 3:**
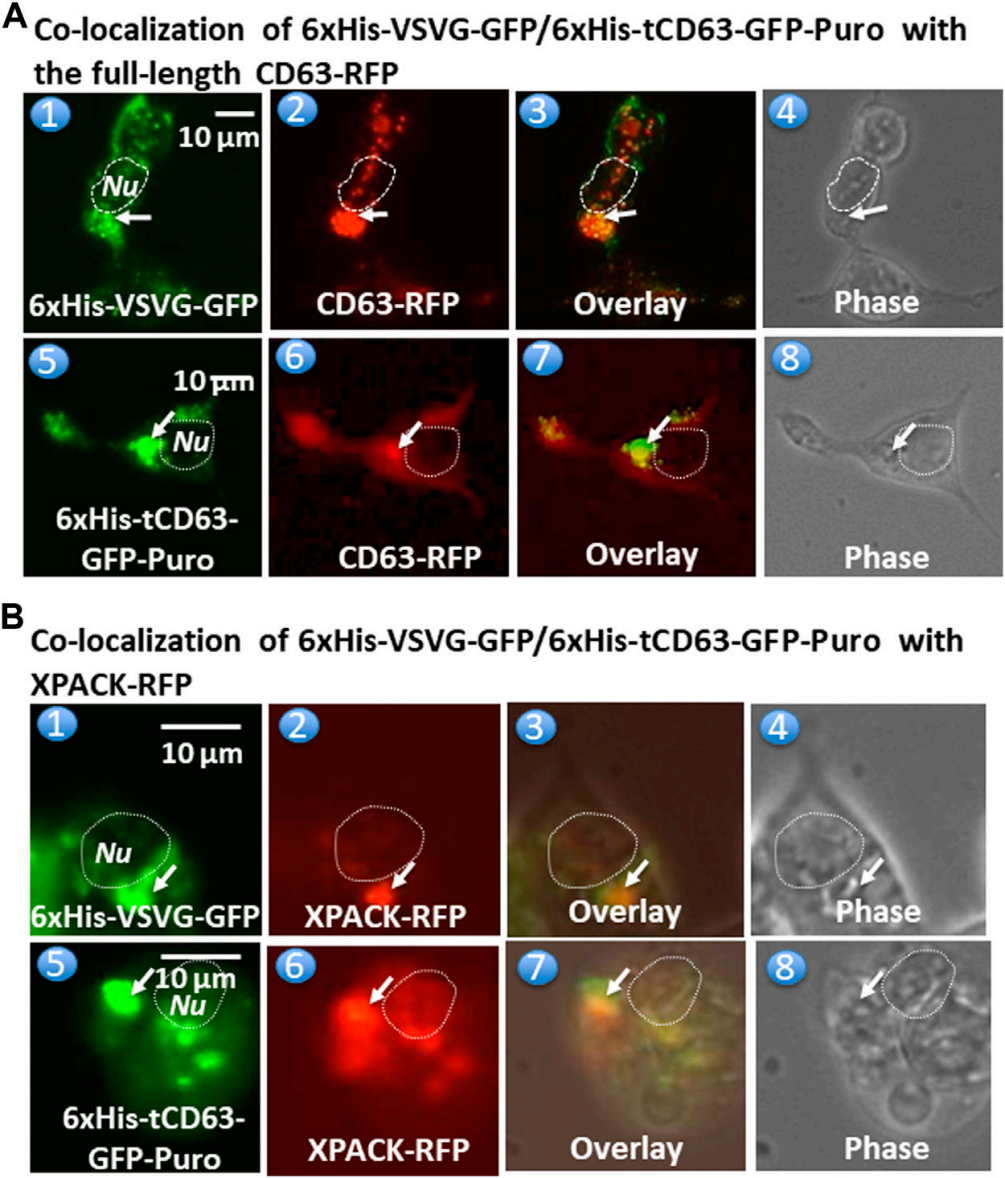
Fusion proteins are targeted and integrated into exosome compartments. 293T cells were transiently transfected with either 6xHis-VSVG-GFP or 6xHis-tCD63-GFP in combination of established exosome marker: the full-length CD63-RFP or XPACK-RFP. Fluorescent and phase contrast images of the same field were recorded on Day 2 post-transfection. Co-localization of either 6xHis-VSVG-GFP (top panel) or 6xHis-tCD63-GFP (lower panel) with full-length CD63-RFP, exhibiting punctates in cytosols. **(A)**. Similar co-localization patterns of either 6xHis-VSVG-GFP (top panels **B**) or 6xHis-tCD63-GFP (lower panels of **B**) with another exosome marker, XPACK-RFP. **(B)**. Arrows indicated endosome/exosome/MVB structures; Dashed circle indicates nucleus (*Nu*). Scale bar, 10 µm.

To further confirm whether these dual-reporters were truly integrated into pre-EV comportments, we conducted co-transfection experiments in 293T cells with either full-length CD63 (a hallmark of exosome) or a commercial exosomal marker, XPACK (Peterson et al., 2015). As expected, while transfection of cells with either 6xHis-VSVG-GFP (Figure 3,A1) or CD63-RFP (Figure 3,A2) alone showed green or red fluorescence signals respectively, co-transfection of both showed some co-localization of punctate patterns in overlaid images (Figure 3,A3). Similar results were obtained from co-transfection study with 6xHis-tCD63-GFP-Puro and CD63-RFP (Figure 3A,5–8). In addition, the expression of 6xHis-VSVG-GFP/6xHis-tCD63-GFP-Puro chimeras was also co-localized with XPACK, a known exosomal marker (Figure 3B). The integration of these dual-reporters into pre-exosomes was a universal phenomenon, since co-transfections using additional cell lines, such as human U87 (Supplementary Figure S2, A1–A8) and mouse L929 cells (Supplementary Figure S2, B1–B8), results in similar co-localizations. Please note that the green or red fluorescence may represent individual particles or their aggregates (interconnected nature of endocytic vesicles), which may not correlate to their true size due to the spatial resolution limit of the fluorescence or confocal microscope (∼225 nm). Although the overlap of GFP and RFP signals are evident in localization experiments, the more accurate quantification may require both advanced imaging system and software support. Together, these results indicate that both the deletion of N-terminal sequences of CD63/VSVG and the addition of a 6xHis tag do not alter the pre-exosomal targeting of our genetic labeling constructs. We conclude that these dual-reporters can be successfully integrated into the pre-exosomal compartments and can be used to monitor exosomal biogenesis pathways in various mammalian cells.

### Preparation, Characterization and Capture of Stably Labeled EVs With Magnetic Beads

**FIGURE 4:**
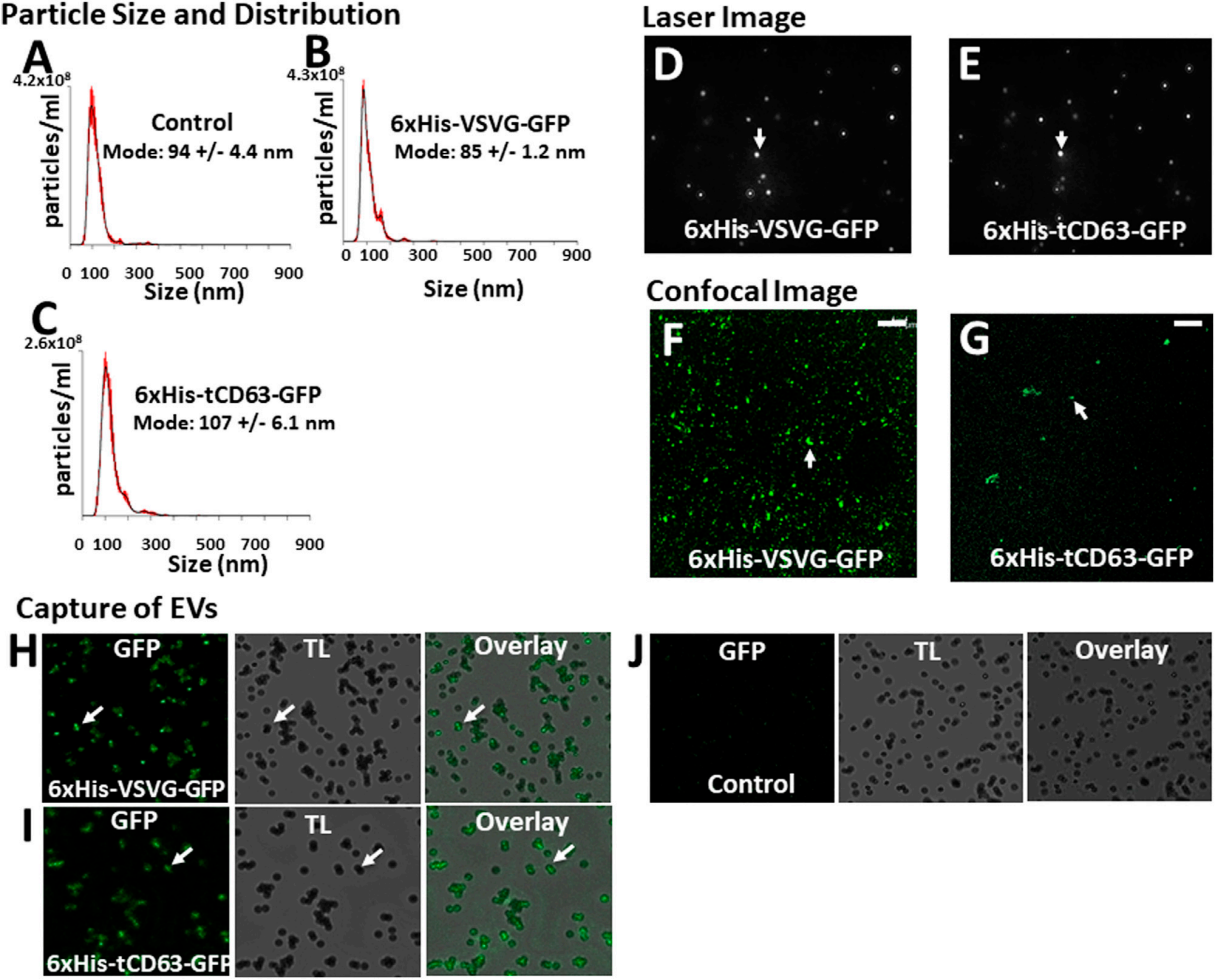
Isolation and characterization of engineered EVs. 72 h post-transfection of 293T cells with either 6xHis-VSVG-GFP or 6xHis-tCD63-GFP-Puro, or non-transfected controls, EVs were isolated and subjected to nanoparticle tracking analysis (NTA) **(A–C)**, laser light reflecting and Brownian motion analysis **(D, E)**, and fluorescent images **(F, G)**. Affinity pull-down of His-tagged EVs using Nickel-coated magnetic beads were conducted and the confocal images of green fluorescence, transmitted light (TL) and overlay, to show specific capture of 6xHis-VSVG-GFP **(H)** and 6xHis-tCD63-GFP **(I)** labelled EVs, but not of non-transfected control EVs **(J)**. Transmitted light (TL). Scale bar, 10 µm.

To determine whether our labelled EVs could mature and ultimately release into the extracellular space, we isolated EVs from the conditioned medium of transfected 293T cells. To focus our study on small EVs (exosomes and microvesicles), we used a combination of ultrafiltration, chemical precipitation and centrifugation procedures as previously reported (Duong et al., 2019). To ensure the purity of EVs, we used serum-free UltraCULTURE medium to exclude the potential contamination of EVs from bovine serum. We then performed a number of characterization studies on isolated EVs collected from both transfected cells and non-transfected controls. First, we verified the particle size and size distribution of the engineered EVs *via* NTA, and found that both 6xHis-VSVG-GFP and 6xHis-tCD63-GFP (Figures 4B,C) exhibited single dominant peak in the range of 80–110 nm without significant differences from the non-modified native controls (Figure 4A). One shall be careful not over interpret the small size differences between the modified and unmodified EVs, which are in the range of 10–15 nm. These differences in mode size may not be biological significant due to technical accuracy of NTA device (Bachurski et al., 2019). Further, we observed typical Brownian motion for both engineered EVs similar to that of non-modified control EVs (Supplementary videos S1–S3), suggesting that different modifications do not affect their physical properties (Figures 4D,E). However, we did not carry out any quantitative analysis on Brownian motion of isolated EVs. Lastly, we confirmed that both chimeric proteins can be successfully integrated into EVs by recording our confocal fluorescence images of isolated EVs preparations (Figures 4F,G). Using chemical defined medium for EVs production, and a combination of centrifugation and ExoQuick precipitation to isolate the EVs, we minimized potential contaminations of both protein aggregates and lipoproteins. The fact that NTA showed a single peak of 80–120 nm in size, and the exhibition of several markers of EVs is assurance (Do et al., 2019; Duong et al., 2019).

An important feature of our engineered EVs is the addition of 6xHis tag to the outer surface of vesicles, thereby allowing affinity capturing with Nickel-beads. To confirm that our isolated GFP-positive EVs could be captured *in vitro*, we co-incubated Nickel-coated magnetic Dyna beads with either 6xHis-VSVG-GFP or 6xHis-tCD63-GFP stably labeled EVs. We found that both modified EVs groups resulted in GFP positivity around the beads under confocal microscopy (Figures 4H,I), presumably due to 6x-His/Nickel binding interactions. In contrast, we could not detect any signals in control groups at the bead surface (Figure 4J), suggesting specific capture of 6xHis-functionalized exosomes not due to background noises. The addition of 6xHis tag to the outer surface of EVs will provide new capabilities for these molecular tools. For example, 6xHis allows specific capturing using nickel-chelation, a widely available technology. This feature, if combined with either flowcytometry (Peterson et al., 2015) or on-chip analysis (Daaboul et al., 2016; Gori et al., 2020), will enable high throughput analysis of engineered EVs, speeding up the clinical translations of EV-based drug delivery and therapy. Whether a dynamic microfluidic system coupled with Ni2+-beads based on the 6xHis tag would eventually obviate ultracentrifugation, ExoQuick and other purifications steps remain to be studied.

### Monitor the Uptake of EVs by Mammalian Cells

**FIGURE 5:**
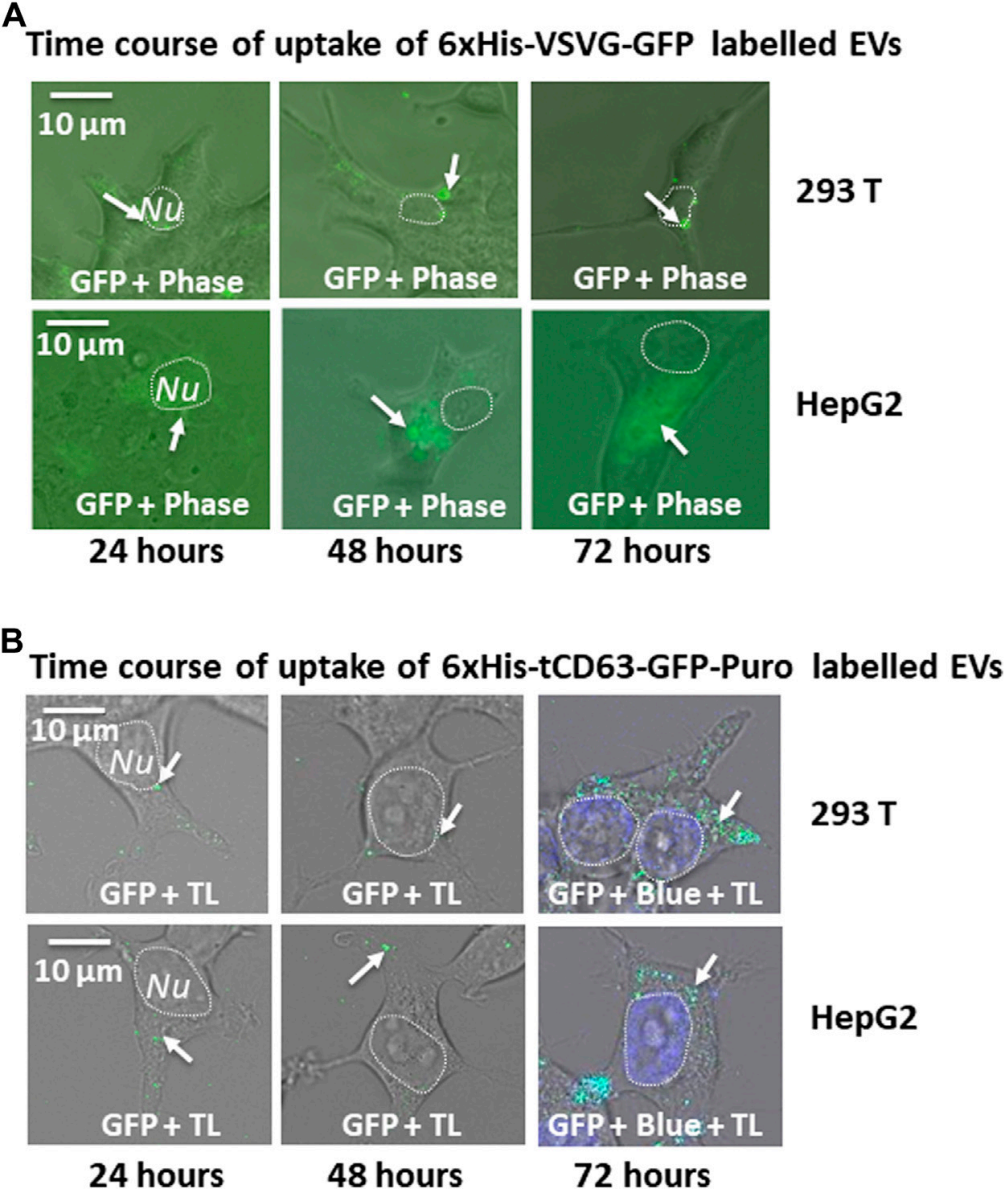
Time-course study of EVs uptake by various mammalian cells. Cellular uptake of dual-tagged EVs in various cultured mammalian cell types. Cultured cells were treated with either 6xHis-VSVG-GFP EVs **(A)** or 6xHis-tCD63-GFP-Puro EVs **(B)** for up to 72 h Images were recorded at indicated time points (24, 48, and 72 h) post-transfection with both fluorescent/confocal and phase contrast/transmitted light (TL) for 293T (right panels) or HepG2 (left panels). Arrows indicate endosome/exosome/MVB structures. Dashed circle indicates nucleus (*Nu*). Scale bar, 10 µm. TL, transmitted light. Blue, DAPI staining.

Another critical application of stably labelled EVs is to monitor their uptake pathway in recipient cells. We next determined whether the uptake of GFP-labelled EVs can be successfully monitored by fluorescent microscopy. First, we conducted a time-course study of co-incubation of 6xHis-VSVG-GFP labeled EVs with two human cell lines (293 T and HepG2). Following the addition of modified EVs to the culture medium, we monitored EVs uptake in those cells up to 72 h by recording fluorescence images using conventional microscope at the indicated time-points (24, 48, and 72 h post-incubation). We found that both cell types exhibited strong discrete GFP punctates in their cytosol as early as 6 h post-EV addition (data not shown), with GFP-positivity steadily becoming evident after 24 h and persisting for at least 72 h (Figure 5A). Similarly, we determined whether 6xHis-tCD63-GFP-Puro labeling can also be used to monitor the uptake of EVs in the same way. We conducted parallel experiments using 6xHis-tCD63-GFP-Puro labeled EVs in 293T and HepG2 cells. In these experiments, we used high definition confocal fluorescence microscope for image recording. As shown in Figure 5B, both 293T and HepG2 cells successfully received GFP-labelled EVs by 24 h post-addition and persisted for at least 72 h, indicating discrete GFP punctates (arrows) in their cytosol, similar to those of 6xHis-VSVG-GFP labelled EVs. Together, these results demonstrated that our stably labelled EVs can be readily uptaken by multiple cell types, a process that can be utilized for real-time monitoring and recording of EVs uptake by either conventional or advanced confocal fluorescence microscopy techniques. The use of stochastic modeling approaches has helped to gain mechanistic insights into organelle biogenesis (Mukherji and O’Shea, 2014; Guzman et al., 2017). Recently, Choubey S et al. propose that a new approach to gain valuable knowledge of organelle biogenesis via computing cell-to-cell variability in organelle abundance at single-cell levels (Choubey et al., 2019). Because our dual-reporters provide additional ability of EV capturing and counting, it is intriguing to see if this new tool can be successfully applied to EV studies.

## Conclusion

We have developed a robust method for stable labelling of EVs by fusing imaging proteins and purification tags to EV-targeting scaffolds. This genetic method enables live cell imaging and affinity-capture of exosomes for the investigation of their biogenesis and uptake in mammalian cells. These molecular tools provide a simple and robust means to study EV biology and to promote advanced EV-engineering.

## Data Availability

The original contributions presented in the study are included in the article/Supplementary Material, further inquiries can be directed to the corresponding authors.

## References

[B1] Bachurski D. Schuldner M. Nguyen P. H. Malz A. Reiners K. S. Grenzi P. C. (2019). Extracellular Vesicle Measurements with Nanoparticle Tracking Analysis - An Accuracy and Repeatability Comparison between NanoSight NS300 and ZetaView. J. Extracell Vesicles 8 (1), 1596016. 10.1080/20013078.2019.1596016 30988894 PMC6450530

[B2] Barile L. Vassalli G. (2017). Exosomes: Therapy Delivery Tools and Biomarkers of Diseases. Pharmacol. Ther. 174, 63–78. 10.1016/j.pharmthera.2017.02.020 28202367

[B3] Choubey S. Das D. Majumdar S. (2019). Cell-to-cell Variability in Organelle Abundance Reveals Mechanisms of Organelle Biogenesis. Phys. Rev. E 100 (2-1), 022405. 10.1103/PhysRevE.100.022405 31574672

[B4] Colombo M. Raposo G. Théry C. (2014). Biogenesis, Secretion, and Intercellular Interactions of Exosomes and Other Extracellular Vesicles. Annu. Rev. Cel Dev. Biol. 30, 255–289. 10.1146/annurev-cellbio-101512-122326 25288114

[B5] Curley N. Levy D. Do M. A. Brown A. Stickney Z. Marriott G. (2020). Sequential Deletion of CD63 Identifies Topologically Distinct Scaffolds for Surface Engineering of Exosomes in Living Human Cells. Nanoscale 12 (22), 12014–12026. 10.1039/d0nr00362j 32463402 PMC7313400

[B6] Daaboul G. G. Gagni P. Benussi L. Bettotti P. Ciani M. Cretich M. (2016). Digital Detection of Exosomes by Interferometric Imaging. Sci. Rep. 6, 37246. 10.1038/srep37246 27853258 PMC5112555

[B7] Do M. A. Levy D. Brown A. Marriott G. Lu B. (2019). Targeted Delivery of Lysosomal Enzymes to the Endocytic Compartment in Human Cells Using Engineered Extracellular Vesicles. Sci. Rep. 9 (1), 17274. 10.1038/s41598-019-53844-5 31754156 PMC6872767

[B8] Duong N. Brown A. Curley K. Brown A. Campanelli A. Do M. A. (2019). Decoy Exosomes as a Novel Biologic Reagent to Antognize Inflammation. Int. J. Nanomedicine 14, 3413–3425. 10.2147/ijn.s196975 31190800 PMC6514129

[B9] Gilkerson R. W. Margineantu D. H. Capaldi R. A. Selker J. M. L. (2000). Mitochondrial DNA Depletion Causes Morphological Changes in the Mitochondrial Reticulum of Cultured Human Cells. FEBS Lett. 474 (1), 1–4. 10.1016/s0014-5793(00)01527-1 10828440

[B10] Gori A. Romanato A. Greta B. Strada A. Gagni P. Frigerio R. (2020). Membrane-binding Peptides for Extracellular Vesicles On-Chip Analysis. J. Extracell Vesicles 9 (1), 1751428. 10.1080/20013078.2020.1751428 32363015 PMC7178839

[B11] Gutiérrez-Vázquez C. Villarroya-Beltri C. Mittelbrunn M. Sánchez-Madrid F. (2013). Transfer of Extracellular Vesicles during Immune Cell-Cell Interactions. Immunol. Rev. 251 (1), 125–142. 10.1111/imr.12013 23278745 PMC3740495

[B12] Guzman H. V. Junghans C. Kremer K. Stuehn T. (2017). Scalable and Fast Heterogeneous Molecular Simulation with Predictive Parallelization Schemes. Phys. Rev. E 96 (5-1), 053311. 10.1103/PhysRevE.96.053311 29347684

[B13] Hung M. E. Leonard J. N. (2015). Stabilization of Exosome-Targeting Peptides via Engineered Glycosylation. J. Biol. Chem. 290 (13), 8166–8172. 10.1074/jbc.M114.621383 25657008 PMC4375473

[B14] Ingato D. Lee J. U. Sim S. J. Kwon Y. J. (2016). Good Things Come in Small Packages: Overcoming Challenges to Harness Extracellular Vesicles for Therapeutic Delivery. J. Control. Release 241, 174–185. 10.1016/j.jconrel.2016.09.016 27667180

[B15] Kalani A. Tyagi A. Tyagi N. (2014). Exosomes: Mediators of Neurodegeneration, Neuroprotection and Therapeutics. Mol. Neurobiol. 49 (1), 590–600. 10.1007/s12035-013-8544-1 23999871 PMC3951279

[B16] Kamerkar S. LeBleu V. S. Sugimoto H. Yang S. Ruivo C. F. Melo S. A. (2017). Exosomes Facilitate Therapeutic Targeting of Oncogenic KRAS in Pancreatic Cancer. Nature 546 (7659), 498–503. 10.1038/nature22341 28607485 PMC5538883

[B17] Kimura S. Noda T. Yoshimori T. (2007). Dissection of the Autophagosome Maturation Process by a Novel Reporter Protein, Tandem Fluorescent-Tagged LC3. Autophagy 3 (5), 452–460. 10.4161/auto.4451 17534139

[B18] Lippincott-Schwartz J. Snapp E. Kenworthy A. (2001). Studying Protein Dynamics in Living Cells. Nat. Rev. Mol. Cel Biol 2 (6), 444–456. 10.1038/35073068 11389468

[B19] Marcus M. E. Leonard J. N. (2013). FedExosomes: Engineering Therapeutic Biological Nanoparticles that Truly Deliver. Pharmaceuticals (Basel) 6 (5), 659–680. 10.3390/ph6050659 23894228 PMC3722064

[B20] Meckes D. G. Jr. Raab-Traub N. (2011). Microvesicles and Viral Infection. J. Virol. 85 (24), 12844–12854. 10.1128/JVI.05853-11 21976651 PMC3233125

[B21] Melo S. A. Luecke L. B. Kahlert C. Fernandez A. F. Gammon S. T. Kaye J. (2015). Glypican-1 Identifies Cancer Exosomes and Detects Early Pancreatic Cancer. Nature 523 (7559), 177–182. 10.1038/nature14581 26106858 PMC4825698

[B22] Meyer C. Losacco J. Stickney Z. Li L. Marriott G. Lu B. (2017). Pseudotyping Exosomes for Enhanced Protein Delivery in Mammalian Cells. Int. J. Nanomedicine 12, 3153–3170. 10.2147/ijn.s133430 28458537 PMC5402897

[B23] Mittelbrunn M. Sánchez-Madrid F. (2012). Intercellular Communication: Diverse Structures for Exchange of Genetic Information. Nat. Rev. Mol. Cel Biol. 13 (5), 328–335. 10.1038/nrm3335 PMC3738855 22510790

[B24] Mukherji S. O'Shea E. K. (2014). Mechanisms of Organelle Biogenesis Govern Stochastic Fluctuations in Organelle Abundance. Elife 3, e02678. 10.7554/eLife.02678 24916159 PMC4046565

[B25] Peinado H. Alečković M. Lavotshkin S. Matei I. Costa-Silva B. Moreno-Bueno G. (2012). Melanoma Exosomes Educate Bone Marrow Progenitor Cells toward a Pro-metastatic Phenotype through MET. Nat. Med. 18 (6), 883–891. 10.1038/nm.2753 22635005 PMC3645291

[B26] Peterson M. F. Otoc N. Sethi J. K. Gupta A. Antes T. J. (2015). Integrated Systems for Exosome Investigation. Methods 87, 31–45. 10.1016/j.ymeth.2015.04.015 25916618

[B27] Petros R. A. DeSimone J. M. (2010). Strategies in the Design of Nanoparticles for Therapeutic Applications. Nat. Rev. Drug Discov. 9 (8), 615–627. 10.1038/nrd2591 20616808

[B28] Pulliam L. Sun B. Mustapic M. Chawla S. Kapogiannis D. (2019). Plasma Neuronal Exosomes Serve as Biomarkers of Cognitive Impairment in HIV Infection and Alzheimer's Disease. J. Neurovirol. 25 (5), 702. 10.1007/s13365-018-0695-4 30610738 PMC7372698

[B29] Ramakrishnaiah V. Thumann C. Fofana I. Habersetzer F. Pan Q. de Ruiter P. E. (2013). Exosome-mediated Transmission of Hepatitis C Virus between Human Hepatoma Huh7.5 Cells. Proc. Natl. Acad. Sci. USA 110 (32), 13109–13113. 10.1073/pnas.1221899110 23878230 PMC3740869

[B30] Riazifar M. Pone E. J. Lotvall J. Zhao W. (2017). Stem Cell Extracellular Vesicles: Extended Messages of Regeneration. Annu. Rev. Pharmacol. Toxicol. 57, 125–154. 10.1146/annurev-pharmtox-061616-030146 27814025 PMC5360275

[B31] EL Andaloussi S. Mäger I. Breakefield X. O. Wood M. J. (2013). Extracellular Vesicles: Biology and Emerging Therapeutic Opportunities. Nat. Rev. Drug Discov. 12 (5), 347–357. 10.1038/nrd3978 23584393

[B32] Shahabipour F. Banach M. Sahebkar A. (2016). Exosomes as Nanocarriers for siRNA Delivery: Paradigms and Challenges. Arch. Med. Sci. 12 (6), 1324–1326. 10.5114/aoms.2016.62911 27904525 PMC5108394

[B33] Shen B. Wu N. Yang J. M. Gould S. J. (2011). Protein Targeting to Exosomes/microvesicles by Plasma Membrane Anchors. J. Biol. Chem. 286 (16), 14383–14395. 10.1074/jbc.m110.208660 21300796 PMC3077638

[B34] Shi J. Heegaard C. W. Rasmussen J. T. Gilbert G. E. (2004). Lactadherin Binds Selectively to Membranes Containing Phosphatidyl-L-Serine and Increased Curvature. Biochim. Biophys. Acta 1667 (1), 82–90. 10.1016/j.bbamem.2004.09.006 15533308

[B35] Simpson R. J. Jensen S. S. Lim J. W. E. (2008). Proteomic Profiling of Exosomes: Current Perspectives. Proteomics 8 (19), 4083–4099. 10.1002/pmic.200800109 18780348

[B36] Sterzenbach U. Putz U. Low L. H. Silke J. Tan S. S. Howitt J. (2017). Engineered Exosomes as Vehicles for Biologically Active Proteins. Mol. Ther. 25 (6), 1269–1278. 10.1016/j.ymthe.2017.03.030 28412169 PMC5474961

[B37] Stickney Z. Losacco J. McDevitt S. Zhang Z. Lu B. (2016). Development of Exosome Surface Display Technology in Living Human Cells. Biochem. Biophys. Res. Commun. 472 (1), 53–59. 10.1016/j.bbrc.2016.02.058 26902116

[B38] Sung B. H. Ketova T. Hoshino D. Zijlstra A. Weaver A. M. (2015). Directional Cell Movement through Tissues Is Controlled by Exosome Secretion. Nat. Commun. 6, 7164. 10.1038/ncomms8164 25968605 PMC4435734

[B39] Tsien R. Y. (1998). The green Fluorescent Protein. Annu. Rev. Biochem. 67, 509–544. 10.1146/annurev.biochem.67.1.509 9759496

[B40] Valadi H. Ekström K. Bossios A. Sjöstrand M. Lee J. J. Lötvall J. O. (2007). Exosome-mediated Transfer of mRNAs and microRNAs Is a Novel Mechanism of Genetic Exchange between Cells. Nat. Cel Biol 9 (6), 654–659. 10.1038/ncb1596 17486113

