# Supplementary material for: Orchestrating Extracellular Vesicle With Dual Reporters for Imaging and Capturing in Mammalian Cell Culture
Source: Front Mol Biosci. 2021 Jun 18;8:680580. doi: 10.3389/fmolb.2021.680580 (PMC8249585; doi:10.3389/fmolb.2021.680580)
Supplement: Supplementary file 1 [file DataSheet2.PDF]

Sequences of dual-reporters

1. 6xHis-VSVG-GFP
2. 6xHis-tCD63-GFP-Puro

==

1. 6xHis-VSVG-GFP

MFMPSSLSYSSWATCWLLCCLIILAKNSSTDPSRN**HHHHHHSDTMKCLLYLAFLFIGVNCKFTIVFPHNQKGNWKNV**
PSNYHYCPSSSDLNWHNDLIGTALQVKMPKSHKAIQADGWMCHASKWVTTCDFRWYGPKYITHSIRSFTPSVEQC
KESIEQTKQGTWLNPGFPPQSCGYATVTDAEAVIVQVTPHHVLVDEYTGEWVDSQFINGKCSNYICPTVHNSTTWH
SDYKVKGLCDSNLISMDITFFSEDGELSSLGKEGTGFRSNYFAYETGGKACKMQYCKHWGVRLPSGVWFEMADKDL
FAAARFPECPEGSSISAPSQTSVDVSLIQDVERILDYSLCQETWSKIRAGLPISPVDLSYLAPKNPGTGPAFTIINGTLKY
FETRYIRVDIAAPILSRMVGMISGTTTERELWDDWAPYEDVEIGPNGVLRTSSGYKFPLYMIGHGMLDSDLHLSSKA
QVFEHPHIQDAASQLPDDESLFFGDTGLSKNPIELVEGWFSSWKSSIASFFFIIGLIIGLFLVLRVGIHLCIKLKHTKKRQI
YTDIEMNRLGKESDESGLPAMEIECRITGTLNGVEFELVGGGEGTPKQGRMTNKMKSTKGALTFSPYLLSHVMGYGFY
HFGTYPSGYENPFLHAINNGGYTNTRIEKYEDGGVLHVSFSYRYEAGRVIGDFKVVGTGFPEDSVIFTDKIIRSNATVEHL
HPMGDNVLVGSFARTFSLRDGGYYSFVVDSHMHFKSAIHPSILQNGGPMFAFRRVEELHSNTELGIVEYQHAFKTPIAF
ARSRAQSSNSAVDGTAGPGSTGSR*

2. 6xHis-Nt-CD63-GFP-Puro

M**HHHHHH**ALVLSQTIIQGATPGSLLPVVIIAVGVFLFLVAFVGCCGACKENYCLMITFAIFLSLIMLVEVAAAIAGYVFRD
KVMSEFNNNFRQQMENYPKNNHTASILDRMQADFKCCGAANYTDWEKIPSMSKNRVPDSCCINVTVGCGINFNEKA
IHKEGCVEKIGGWLRKNVLVVAAAALGIAFVEVLGIVFACCLVKSIRSGYEVMMESDESGLPAMEIECRITGTLNGVEFEL
VGGGEGTPKQGRMTNKMKSTKGALTFSPYLLSHVMGYGFYHFGTYPSGYENPFLHAINNGGYTNTRIEKYEDGGVLH
VSFSYRYEAGRVIGDFKVVGTGFPEDSVIFTDKIIRSNATVEHLHPMGDNVLVGSFARTFSLRDGGYYSFVVDSHMHFKS
AIHPSILQNGGPMFAFRRVEELHSNTELGIVEYQHAFKTPIAFARSRAQSSNSAVDGTAGPGSTGSRHMTEYKPTVRLA
TRDDVPRAVRTLAAAFADYPATRHTVDPDRHIERVTELQELFLTRVGLDIGKVWVADDGAAVAVWTTPESVEAGAVF
AEIGPRMAELSGSRLAAQQQMEGLLAPHRPKEPAWFLATVGVSPDHQGKGLGSAVVLPGVEAAERAGVPAFLETSAP
RNLPFYERLGFTVTADVEVPEGPRTWCMTRKPGA*

The color scheme is used for annotation: Yellow: Signal peptides; Purple: 6xHis; Uncolored: **VSVG**; Green: GFP; Gray: Puro; Red: RFP; tCD63: N-terminal transmembrane domain deleted CD63.
