# Supplementary material for: Orchestrating Extracellular Vesicle With Dual Reporters for Imaging and Capturing in Mammalian Cell Culture
Source: Front Mol Biosci. 2021 Jun 18;8:680580. doi: 10.3389/fmolb.2021.680580 (PMC8249585; doi:10.3389/fmolb.2021.680580)
Supplement: Supplementary file 4 [file DataSheet1.PDF]

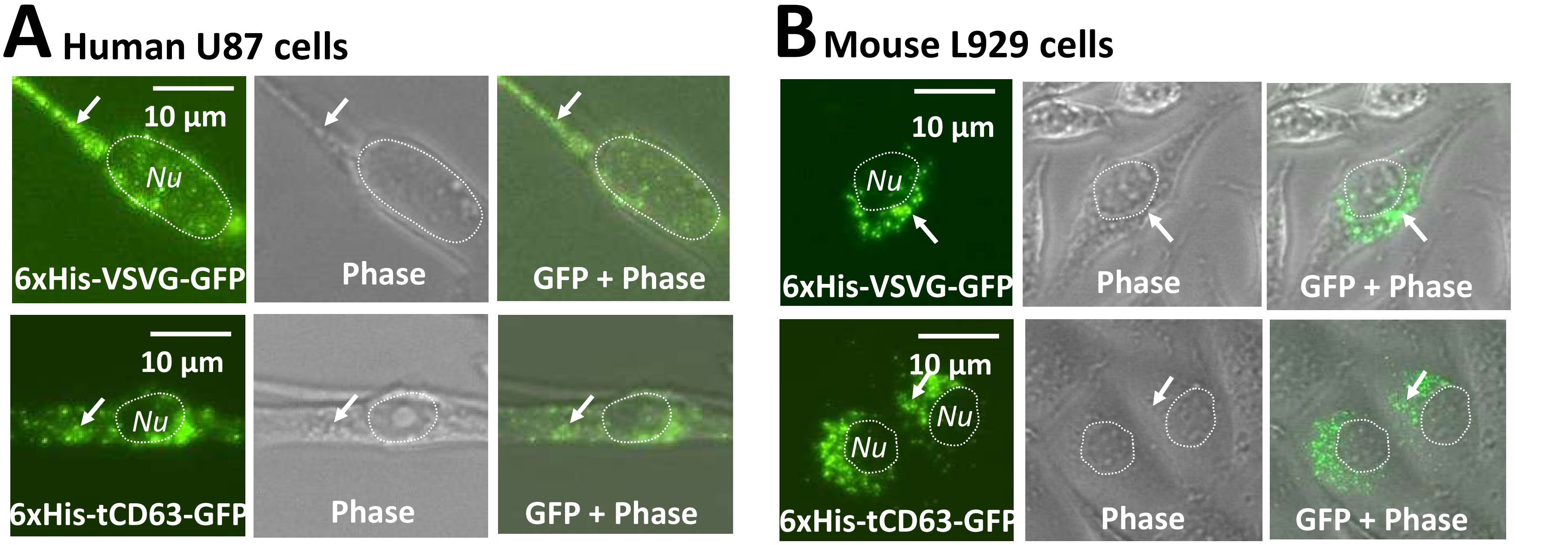

Supplementary Figure S1. Live imaging of expression of chimera proteins in human U87 and mouse L929 cells. U87 (**A**) and L929 (**B**) were transfected with either 6xHis-VSVG-GFP (upper panels) or 6xHis-tCD63-GFP (lower panels) fusion genes and imaged on Day 2 post-transfection. Green fluorescence signals (arrows) showed in punctate pattern within the cytosol, indicating the expression of chimera proteins in transfected cells. *Nu*: nucleus. Arrows indicate endosome/exosome/MVB structure. Scale bar 10 µm.

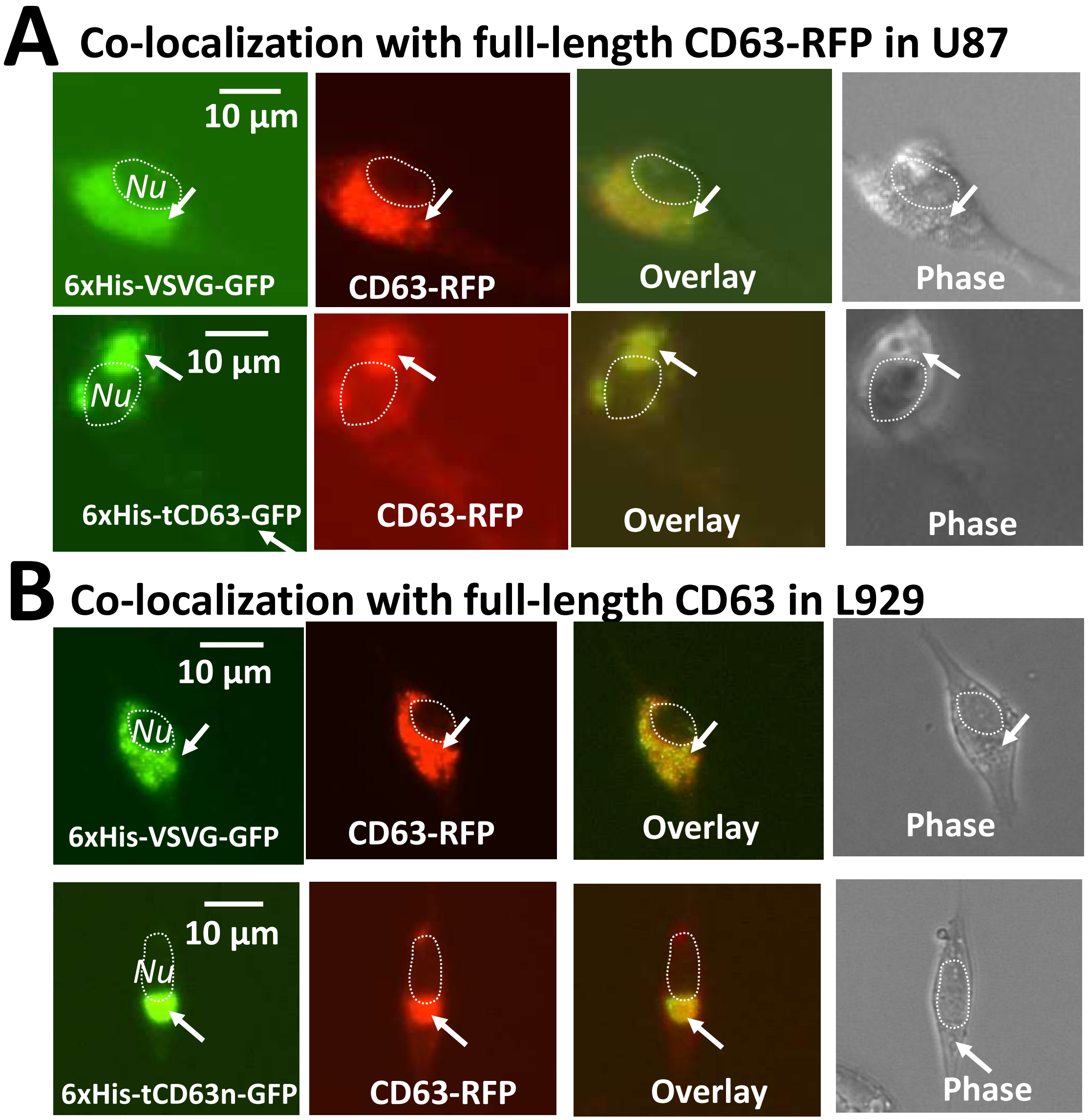

Supplementary Figure S2. Co-localization of the dual-tagged fusion proteins with the full-length CD63-RFP in human U87 (A) and mouse L929 (B) cells. Human U87 (**A**) and mouse L929 (**B**) were co-transfected with either 6xHis-VSVG-GFP (top panels) or 6xHis-tCD63-GFP (bottom panels) and the full-length CD63-RFP for 2 days. Images of fluorescence signals and phase contract of the same field were recorded to demonstrate the same subcellular localizations (yellow color). *Nu*: nucleus. Arrows indicate endosome/exosome/MVB structure. Scale bar 10 µm.
